# Energy-Efficient Cluster Management Using a Mobile Charger for Solar-Powered Wireless Sensor Networks

**DOI:** 10.3390/s20133668

**Published:** 2020-06-30

**Authors:** Youngjae Son, Minjae Kang, Younghyun Kim, Ikjune Yoon, Dong Kun Noh

**Affiliations:** 1 Department of Software Convergence, Soongsil University, Seoul 06978, Korea; karit@ssu.ac.kr; 2 Department of Electronic Engineering, Soongsil University, Seoul 06978, Korea; minjaekang@ssu.ac.kr; 3 Department of Electrical and Computer Engineering, University of Wisconsin, Madison, WI 53706, USA; younghyun.kim@wisc.edu; 4 Department of Smart Systems Software, Soongsil University, Seoul 06978, Korea; ijyoon@ssu.ac.kr

**Keywords:** wireless sensor network, mobile sink, wireless power transmission, clustering

## Abstract

In solar-powered wireless sensor networks (SP-WSNs), sensor nodes can continuously harvest energy to relieve the energy constraint problem in battery-powered WSNs. With the advent of wireless power transmission (WPT) technology, the nodes can be charged remotely if the energy harvested is insufficient. However, even in SP-WSNs with WPT, an energy imbalance problem is observed, in which the energy consumption of the nodes around a sink node increases abnormally if the sink node is stationary. To solve this problem, recent studies have been conducted using a mobile sink node instead of a stationary one. Generally, a clustering scheme is used for the efficient utilization of a mobile sink. However, even in the case of mobile sinks, it is still necessary to minimize the energy burden of the cluster heads and their surrounding nodes. In this study, we propose a scheme that mitigates the energy imbalance problem of SP-WSNs by using a WPT-capable mobile sink and an efficient clustering scheme. In the proposed scheme, the energy imbalance is minimized by electing the cluster heads effectively after considering the energy state of the nodes, and by enabling the sink node to charge the energy of the cluster heads while collecting data from them. Consequently, this scheme allows the sink node to collect more data with fewer blackouts of the sensor nodes.

## 1. Introduction

Wireless sensor networks (WSNs) deploy numerous low-power and low-cost sensor nodes to monitor the environment in difficult-to-access or vast areas. Recently, WSNs have been widely used in several fields, such as the military, disaster detection, habitat monitoring, and smart homes [1,2]. However, the restricted operation time of WSN nodes is a problem resulting from their limited battery capacity, and accordingly, various studies have been conducted to reduce their operational energy consumption [3]. In addition, the transmission range of a node is relatively short owing to the use of low-cost sensor nodes, and WSNs generally use a stationary sink node. Therefore, the nodes adopt a multi-hop approach that forwards data to the stationary sink node through other nodes. However, this approach can lead to hotspot problems (i.e., traffic concentrations), where the nodes surrounding the sink node can consume a relatively large amount of energy due to the heavy relay traffic [4].

Several studies have been conducted to solve these problems. As the foremost solution, energy harvesting WSNs, which use nodes that collect energy from the environment, are deployed to fundamentally solve the energy constraint problem of battery-powered sensors [5,6]. Solar energy is preferred among the various environmental energy sources due to its periodicity and high power density. In addition, wireless power transmission (WPT) for WSNs using mobile chargers was proposed in [7]. In this case, the mobile charger can visit and charge only a small number of nodes due to the limited energy in mobile devices. Accordingly, numerous studies have been undertaken to determine the optimal nodes to be charged and discover the best paths to these nodes [8].

Research has also been conducted on mitigating the energy imbalance problem of the nodes surrounding the stationary sink. Among them, the idea of using a mobile sink has been the most actively studied [9]. However, in this case, an incomplete collection of data is a problem, as the mobile sink cannot visit all the sensor nodes for collection owing to its limited energy. Therefore, in most studies, a cluster-based data collection scheme was used to reduce the moving distance of mobile sinks [10]. In this approach, the sink node needs to visit only limited number of nodes, called cluster heads, to collect data. However, even with the cluster-based approach, the energy shortage problem in the cluster heads and their surrounding nodes cannot be avoided. In cluster-based topology, moreover, the problems of searching for the optimal clustering and cluster heads to balance the energy consumption are considered to be non-deterministic polynomial-time hard (NP-hard) problems, and thus, have no exact solution [11,12]. Therefore, a heuristic algorithm is needed to solve the problems. In this study, we used a harmony search algorithm (HSA-WSN) [13] as a meta–heuristic algorithm.

In this study, based on the studies described above, we proposed an efficient cluster management scheme designed for solar-powered wireless sensor networks (SP-WSNs) operating with a WPT-capable mobile sink. First, the proposed scheme performed with close-to-optimal clustering for SP-WSNs using the harmony search algorithm. Additionally, in our scheme, the mobile sink charged the cluster heads using WPT, thereby relieving their energy burden. Lastly, energy hotspots in the nodes close to the cluster heads were reduced by using an effective head selection algorithm, which led to a decrease in the blackout times of the sensor nodes and an increase in the amount of data collected at the mobile sink. The main contributions of this work are as follows.

- **Accomplishment of near-optimal clustering**: Finding the optimal cluster topology is considered to be an NP-hard problem. However, our scheme provides a close-to-optimal clustering of the SP-WSN through a harmony search algorithm, which is a simple but effective meta-heuristic algorithm. Our cluster-head-selection scheme operated based on this cluster topology as determined by the harmony search algorithm.
- **Settlement of energy shortage problem in cluster heads**: Even if solar energy is harvested, the energy consumption of the cluster head is considerable. The mobile charger ensures the best energy distribution to each cluster head based on the amount of energy required for each cluster head, the amount of energy it owns, and the length of the travel path. Through this, the problem of the energy shortage of the cluster head can be solved.
- **The solution of the energy-hotspot problem**: The cluster head receives energy through a mobile charger, but nodes near the cluster head do not receive energy despite using a relatively large amount of energy. In order for these nodes to recover energy, the energy consumption during the next round should be adjusted so that the amount of energy consumed by these nodes is less than the amount of harvested energy. Our scheme selects the next round of cluster heads delicately so that these nodes can minimize their energy consumption. This allows energy-hotspot nodes to sufficiently recover energy.
- **Achievement of high performance**: Finally, the energy balance between nodes can be achieved from the contributions mentioned above. Therefore, the number of blackout nodes becomes small, so that the network can be operated stably and the amount of energy collected by the sink also can be increased.

The proposed scheme can be applied to WSN applications that are deployed at the locations where human access is difficult or human infrastructure is scarce in the vicinity; for example, forests, deserts, glaciers, volcanoes, and battle fields. In such locations, solar energy harvesting is an attractive option with which to prolong the WSN lifetime and to possibly enable perpetual operation. However, the availability of energy sources may be inconsistent across the deployment area, creating an energy imbalance. The non-uniform workloads performed by each node further aggravate this energy imbalance. In this situation, a mobile charger using WPT can be used to balance the energy provisioning by distributing energy efficiently to the energy-scarce nodes. Without applying a mobile charger, solar-powered nodes that are blacked out for a certain period of time can occur frequently, so the quality of service (QoS) of the WSN application can be lowered. Our scheme aimed to reduce the number of blackout nodes and to maximize the amount of data collected in the sink by minimizing the energy imbalance in the SP-WSN operating with a mobile charger.

The remainder of this paper is organized as follows. In Section 2 we present the existing schemes related to energy harvesting WSNs and WPT, and meta-heuristic algorithms for creating a cluster and electing a cluster head. In Section 3, we detail the proposed scheme, and in Section 4, we show the performance evaluation of the proposed scheme through experiments. Lastly, in Section 5, we conclude this paper with a brief summary of the study.

## 2. Related Work

### 2.1. Energy-Rechargeable WSNs

The majority of studies related to battery-based WSNs have been conducted to increase the lifetime of the network by minimizing the energy consumption. However, the lifetime still remains limited due to the inherent limitations of the battery capacity. In such a case, the battery should be periodically charged or replaced, which is not a major problem in applications such as smart homes, where we can easily access the sensor devices. However, in applications such as those in military areas or for ecosystem monitoring, sensors are occasionally installed in difficult-to-access places, making it expensive or impossible to replace the batteries due to the nature of the environment. To address this problem, energy harvesting WSNs and WPT have been actively researched as a solution.

#### 2.1.1. Energy Harvesting Wireless Sensor Node

An energy harvesting wireless sensor node charges its battery by harvesting energy from the environment and converting it into electrical energy, and can thereby operate indefinitely in the absence of any other hardware failure. As shown in Table 1, a few possible environmental energy sources are solar, wind, vibration, and pressure [14]. Among them, solar energy is the most widely used in WSNs owing to its high power density, periodicity, and predictability.

Notably, (1) minimization of the energy consumption is crucial in battery-based WSNs, whereas the best use of the harvested energy (maximizing the energy utilization) is most important in SP-WSNs. As the energy is continuously harvested in SP-WSNs, if the focus remains only on minimizing the energy consumption, the surplus harvested energy that exceeds the battery capacity can be wasted (cannot be stored in the battery). (2) A node should not consume more energy than the amount of energy collected. It is important to control the energy consumption rate based on the amount of the harvested energy. This is called an energy-neutral operation (ENO). The battery becomes exhausted and remains in the blackout state for a long time if ENO is not achieved [15]. Therefore, there has been active research on maximizing the energy utilization while satisfying ENO conditions [16,17].

#### 2.1.2. Wireless Power Transfer (WPT)

WPT technology, used to transmit power wirelessly, was successfully attempted by a scientist named Nicola Tesla in the early 20th century, and has gained popularity with the advent of magnetic resonance technology. The technology is used in various applications, such as mobile communication devices, electric vehicles, and WSNs. In particular, for WSNs, automobiles [18] or unmanned aerial vehicles (UAVs) [19] equipped with WPT devices are used to charge sensor nodes and collect data while moving. As shown in Table 2, although energy is relatively infinitely available for automobiles, there must be roads for them to travel. On the other hand, drones, a type of UAV, can reach the sensor nodes by moving through the air without the need for roads. However, drones also have a limited range of flight owing to battery limitations. Therefore, studies have been conducted to discover an efficient traveling path for the mobile sink and to determine the optimal amount of energy to be transferred to each sensor node along that path [20].

### 2.2. Clustering for a Mobile Sink Node

A mobile sink node can visit only a certain number of nodes due to its limited moving distance, which necessitates the application of clustering technology. If there is a head node that can take charge of collecting the data for each cluster, the mobile sink can then visit only these cluster head nodes. As there is no known deterministic polynomial time algorithm that can be used to configure the optimal cluster for a network, meta-heuristic algorithms are often used for solving this problem instead [11,12].

This method gradually finds the close-to-optimal solution by imitating the phenomenon of increasing stability or adaptability through repetition over several years, similarly to evolution in nature. For example, a genetic algorithm [21] imitates the manner in which an organism evolves while adapting to its environment; a particle swarm optimization (PSO) [22] algorithm imitates the behavioral characteristics of creatures that exhibit collective behaviors, such as birds and fish; and a harmony search algorithm (HSA) [23] imitates the process of finding harmony that produces beautiful sounds when composing music. Although these schemes have different characteristics, they are theoretically simple. Therefore, they are used for optimization purposes, not only in engineering but also in fields such as natural science and business administration. In this study, among the various available meta-heuristic algorithms, we tailored the HSA for WSNs (HSA-WSN) [13] owing to the simple implementation and easy application for WSNs. This algorithm finds the clusters and cluster heads by considering the cohesion between the sensor nodes and the distance between the clusters. We describe the operational methodology of the HSA-WSN in Section 3.2.

### 2.3. Cluster Head Selection Method

There are three main methods for selecting the cluster heads (CHs): (1) random selection, (2) a selection method considering the residual energy, and (3) a selection method considering multiple factors, including the energy and location information.

The low energy adaptive clustering hierarchy (LEACH) scheme [24] is a typical protocol among random selection methods that uses probabilities within a cluster. The cluster topology is reconstructed by randomly selecting the CHs for a specific round. LEACH involves two phases in each round: the set-up phase and the steady phase. The set-up phase stochastically selects the head node (once selected, that node is not selected again). In the steady phase, the member nodes transmit their data to the CH according to the scheduling of each cluster, and the CH merges the data and transmits them to the base station.

Examples of the second method which take into consideration the residual energy of the nodes, are the hybrid energy-efficient, distributed clustering (HEED) [25] scheme and the energy-LEACH (E-LEACH) [26] scheme, which improves upon the LEACH method. When E-LEACH selects the head, this method considers only the amount of residual energy, and thus the node with the most residual energy is selected as a head node. Compared with E-LEACH, HEED is a single-hop clustering protocol that considers both the residual energy and the node proximity when selecting CHs. First, it selects the node with the largest amount of residual energy as the head node. However, in case of nodes with similar energy values, it compares their distance with all the neighboring nodes in the cluster and selects the node with excellent proximity to its neighbors as the head.

Lastly, we explain several examples of the third method that select CHs, considering multiple factors, including the energy and location information. HEEL [27] selects CHs in consideration of four parameters per round: the node energy, energy of neighboring nodes, number of hops to a base station, and number of neighboring nodes. Each of these parameters has an impact in selecting the CH. The node with the largest sum of these four values (weights applied) is selected as the head.

MW-LEACH [28] selects CHs based on the residual energy, the distances between CHs, and an optimal number of member nodes. The nodes are selected from the initial set based on the high residual energy closer to the center of the density, thereby forming an initial set of CH candidates. The candidates then move in different directions to collect data from their members and send the data to the base station.

PSO-EC [29] calculates the energy distribution by fixing the coverage area and then selecting the energy centers as CHs. By selecting the node with the highest energy among the surrounding nodes as CH, the energy efficiency is improved. During the first period, this method used the geometric method to select the CHs, and the topology was maintained for several rounds. After the energy of the network became heterogeneous, the special clustering using PSO (particle swarm optimization) was executed to search the energy centers for CH elections.

When using clustering based on PSO-EC, a specific location is first determined for CH. Then, the node closest to this location is defined as CH. To avoid this unnecessary computation in the first period, SMOTECP [30] was proposed, in which the CH selection was directly optimized. A binary SMO (spider monkey optimization) was adopted by considering the CH selection as a binary problem where the CH nodes were labeled as 1 and the others as 0 for optimization using Boolean operations. However, this method cannot control the number of CHs, because the Boolean operations retrieve varying numbers of them (i.e., CHs), which can in turn affect the fitness values and undermine the optimization. In addition, SMOTECP is difficult to apply in networks where the number of CHs is important.

SSMOECHS [31] was proposed as a sampling-based SMO method. If the sampling population consists of nodes to select cluster-heads, the cluster-heads are selected among those nodes. As a result, problems such as increased computation, poor selection accuracy, and the selection of duplicate nodes, are resolved.

However, all the schemes described above were designed for neither solar-powered sensor nodes nor mobile chargers. Their purpose was to reduce the number of dead nodes and increase the lifetimes of battery-based wireless sensor networks.

## 3. Efficient Cluster Management Using a Mobile Charger in SP-WSNs

**Figure 1:**
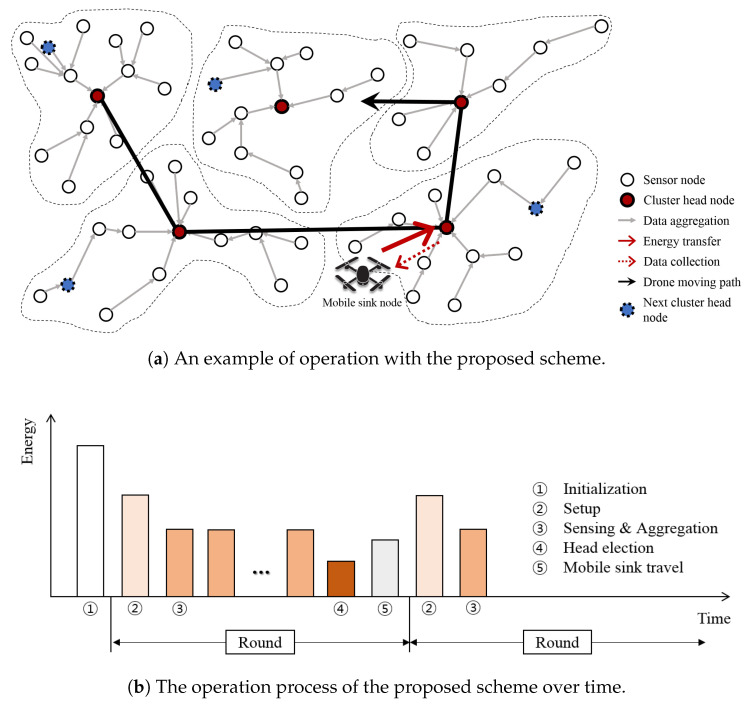
Overview of the proposed scheme.

In this section, we describe the proposed scheme in detail, and an overview of the scheme is also presented in Figure 1. A brief working of the scheme is as follows.

(1) First, in the initialization phase (denoted as ① in Figure 1b), the network is divided into clusters using the meta-heuristic algorithm shown in Figure 1a, and the head of each such cluster is selected, denoted by a red node in the figure. In the setup phase, (denoted by ② in Figure 1b) a path tree is constructed over which the member nodes in the cluster can transmit the collected data to the cluster head. In the subsequent phases, each node starts collecting the data. The details are described in Section 3.2.

(2) Assuming that each cycle of the mobile sink visiting the cluster head comprises one round, then in each cluster, the node that will act as the cluster head for the next round should be selected (denoted by ④ in Figure 1b) before the mobile sink arrives for the current round (denoted by ⑤ in Figure 1b). The blue nodes in Figure 1a indicate the cluster heads that are selected for the next round. There must be a strategy to select the heads for the next rounds by taking into account various factors, such as the configuration of the nodes in the cluster, to minimize the energy imbalance around the current cluster head nodes. The detailed algorithm for electing the next head is described in Section 3.3.

(3) At the end of the round (the mobile sink travel phase denoted as ⑤ in Figure 1b), in addition to electing the head for the next round, the mobile sink visits the current cluster heads, as shown by an arrow in Figure 1a, to collect data and simultaneously transmit an appropriate amount of energy to the head. Even though the head node is a solar energy harvesting node, it consumes considerable energy owing to its role as the cluster head. Therefore, it must be charged to allow it to operate as a normal sensor node for a certain duration. The method to determine the amount of energy to be charged to each head node is described in detail in Section 3.4. In this manner, the mobile sink can collect the data and provide an energy recharge while visiting the current cluster heads. During this trip, the sink can also identify the heads for the next round (which are already selected), and can visit them at the end of the next round.

### 3.1. Energy Neutral Operation (ENO) of Solar-Powered Nodes

Generally, a solar-powered sensor node controls its system parameters (such as the duty cycle, sensing frequency, and transmission range) to continuously ensure a lower energy consumption than the amount charged. Therefore, the residual energy $E_{\mathrm{residual}}$ generally increases monotonically over time, and results in its not becoming blacked out [15]. The operation with this restriction is called an ENO.

As the solar-powered sensor node can satisfy ENO by controlling various system settings, stable operation is ensured. However, the cluster heads cannot satisfy ENO, even if all other usage is reduced to a minimum, as they inevitably use a high amount of energy to fulfill the role of a header node. Therefore, the mobile sink should charge the cluster heads with energy while visiting them to collect data to guarantee the ENO of the cluster heads.

Notably, the mobile sink only charges the cluster head nodes, and the nodes in the hotspot area around the cluster heads do not get charged by the mobile sink. However, it is clear that they also consume more energy than other sensor nodes due to being used for relaying data during the current round and do satisfy ENO at the end of the round. Therefore, in the next round, it is necessary that their available energy is restored (by reducing the consumption of energy) to satisfy the ENO requirements as a whole, barring which, they may eventually black out at some point in time. Consequently, the selection of the next cluster heads is crucial. For example, if there is a substantial pre-existing energy imbalance in a certain cluster, and the location of the next selected cluster head is also close to the current head, the current hotspot is likely to remain a hotspot in the next round also, leading to blackouts in those nodes.

To prevent this occurrence, the next cluster head should be determined in such a way that the nodes currently in the hotspot should not be included in the hotspot area in the next round. This will allow the nodes in the current hotspot area to be charged to the maximum possible in the next round, such that they eventually meet the ENO requirements. In the proposed scheme, we ensure that ENO is guaranteed in the long term by considering this factor while electing the next cluster head.

### 3.2. Initial Clustering

**Figure 2:**
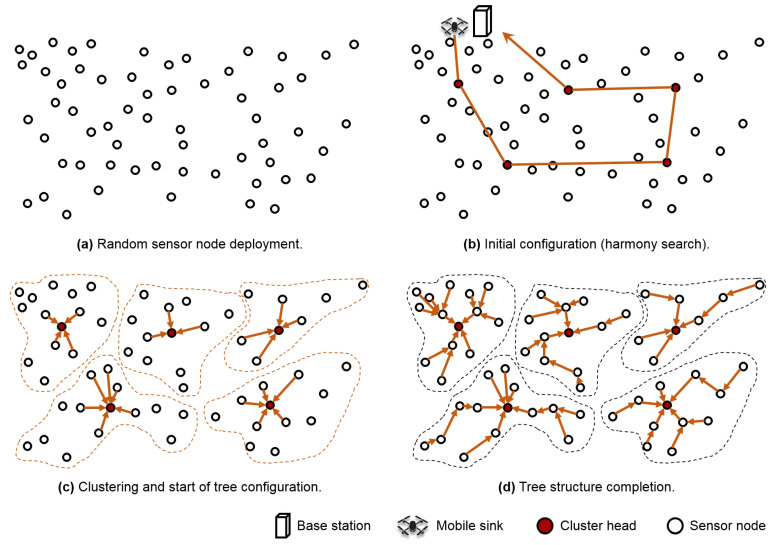
The initial clustering configuration.

Figure 2 shows a simple overview of the clustering process after the initial deployment of wireless sensor nodes. For close-to-optimal clustering in a randomly deployed WSN, as seen in Figure 2a, the base station uses the HSA based on the location information of the sensor nodes. An HSA can determine the appropriate number of clusters as well as the cluster head and members of each cluster. To find such clusters, this method first determines the value of the objective function. The function identifies whether a cluster has the appropriate configuration by considering the cohesion between the cluster members and the distance between the clusters. This objective function provides the minimum value when there is minimum cohesion between the cluster members and the maximum distance between the clusters. In this study, the objective function XB, known as the Xie–Beni (XB) index [32], is represented as follows:

(1) $$\begin{array}{cc} XB & =\frac{\Sigma_{j=1}^{c}\Sigma_{i=1}^{N}\mu_{ij}^{2}{∥n_{i}-{CH}_{j}∥}^{2}}{N({min}_{i,j}\left({∥{CH}_{i}-{CH}_{j}∥}^{2}\right))},\\ \mu_{ij} & =\frac{1}{\sum_{k=1}^{c}{\left(\frac{∥n_{i}-{CH}_{j}∥}{∥n_{i}-{CH}_{k}∥}\right)}^{2}}. \end{array}$$

Here, *c* is the total number of cluster heads, *N* is the total number of sensor nodes, $n_{i}$ is the *i*th sensor node position, and CH is the candidate cluster head location. $∥\cdot ∥$ is the usual Euclidean norm. Thus, $∥n_{i}-{CH}_{j}∥$ is just the Euclidean distance between $n_{i}$ and ${CH}_{j}$. The degree of membership $\mu_{ij}$ refers to the degree of proximity of each sensor node ($n_{i}$) with the candidate head (${CH}_{j}$) and has a value between 0 and 1. The objective function XB represents the ratio of the sum of the distances between the clusters to the cohesion between the cluster members, calculated by using the degree of membership. Therefore, a close-to-optimal cluster can be obtained by selecting the cluster with the minimum value of XB.

The HSA operation is accomplished as follows. The algorithm starts by randomly determining multiple possible solutions (i.e., a list of candidate cluster heads). Next, it finds the best solution among them by evaluating these candidates using the objective function described earlier. This technique then continuously adjusts the best solution until the close-to-optimal solution is achieved. Two methods are used for adjustment: memory recall and pitch adjustment. Memory recall is used to select the better choice between the current best solution and a randomly determined new solution. The pitch adjustment method changes the head nodes in the current best solution with the sensor nodes located closest to them, and compares the resulting solution with the current best solution. By iterating this calculation process, HSA can eventually find the close-to-optimal solution that has the smallest XB. Consequently, this algorithm can also determine the close-to-optimal numbers of clusters, cluster heads, and cluster members.

In addition, the mobile sink that acquires the location information of the head nodes selected by HSA can calculate the shortest path to all the cluster heads and the amount of energy that can be distributed to each of them. As shown in Figure 2b, the mobile sink visits the cluster heads using this information, and informs them about their selection as the heads, and also about the nodes selected as the cluster members. Simultaneously, it charges the heads with extra energy, divided equally according to the number of clusters. The cluster head uses flooding to perform the clustering with the information received from the mobile sink. For clustering, a tree structure is constructed by selecting the sender of the first-arriving packet as the parent of the node, as seen in Figure 2c. Finally, the nodes can start collecting data once the tree structure has developed completely as shown in Figure 2d.

### 3.3. Cluster Head Election

Before the mobile sink reaches the cluster head at the end of a round, each node sends information regarding its available energy and the number of neighboring nodes to the current cluster head. The cluster head selects a few candidate nodes (from among all member nodes) as the next-round cluster head if their residual energy satisfies the following condition:

(2) $$\bar{E_{\mathrm{header}}^{\mathrm{extra}}}+\bar{E_{\mathrm{consume}}}<E_{\mathrm{residual}}.$$

Here, $\bar{E_{\mathrm{header}}^{\mathrm{extra}}}$ is the average energy consumed for functioning as the cluster head of this particular cluster (in addition to that consumed for operating as a normal member node) and $\bar{E_{\mathrm{consume}}}$ is the average energy consumed by the member nodes in this cluster.

To select a cluster head among these candidate nodes, the cluster head calculates the objective function $R_{i}$, and selects the node with the highest $\bar{E_{\mathrm{consume}}}$ as the next cluster head. $R_{i}$ is calculated as follows:

(3) $$R_{i}=\alpha \left(1-\frac{{EV}_{i}}{{EV}_{\max}}\right)+\beta \frac{{NN}_{i}}{{NN}_{\max}}+\gamma \frac{{HC}_{i}}{{HC}_{\max}}.$$

Here, *i* is the index of candidate heads, and $EV_{i}$, $NN_{i}$, and $HC_{i}$ are the energy variance between the node *i* and the neighboring nodes, the number of neighboring nodes of node *i*, and the number of hops between the current head and node *i*, respectively. ${EV}_{\max}$, ${NN}_{\max}$, and ${HC}_{\max}$ are the largest values among $EV_{i}$s, $NN_{i}$s, and $HC_{i}$s, respectively. α, β, and γ are the weighting factors, and are described in Table 3.

The candidate heads with small $EV_{i}$ values indicate that their energy imbalance with the neighboring nodes is not significant, and that adequate energy consumption was achieved in the current round. Additionally, $NN_{i}$ should be considered for calculating $R_{i}$ as a candidate node located at the end of the tree or with a small number of neighboring nodes can be selected as the head only if the EV is considered. In such a situation, when it becomes the next head, the nodes around it can become severe hotspots. Lastly, HC is used to find the candidate head node that is properly spaced and situated in places where the energy consumption can be balanced. As this value grows, it implies that the distance between node *i* and the current head is also increasing. Accordingly, the nodes that were in the hotspot area in this round can recover substantial energy in the next round. The values of α, β, and γ can be determined experimentally, out of which α is the most crucial. The effects of these three parameters are described in more detail in Section 3.6.

Once the election of the head is completed, the current cluster head informs the elected node that it is the head for the next round, and delivers $\bar{E_{\mathrm{header}}^{\mathrm{extra}}}$ to the selected head node. $\bar{E_{\mathrm{header}}^{\mathrm{extra}}}$ is updated continuously using the moving average for each cluster, and is used as an indicator of how much extra energy should be distributed from the mobile sink to each cluster head.

After the mobile sink has visited the current cluster head to collect the data, transmit energy, and gather information on the next head node, the next round is initiated. At the beginning of the next round, the newly elected cluster head reconstructs the tree using a flooding control message. Although the tree is configured similarly as it is in the initial phase, the difference is that the tree structure does not change after receiving a flooding message from the same parent node in the previous round, and thus the flooding is stopped to minimize further energy waste.

**Figure 3:**
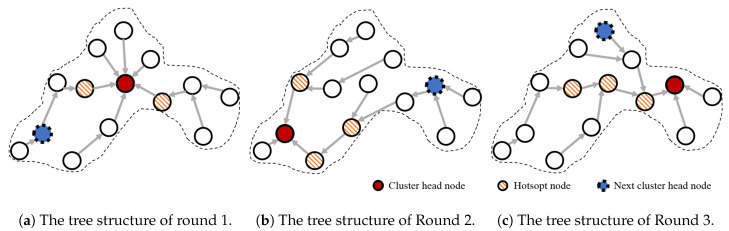
Changes in the tree structure within a cluster.

Figure 3 shows the changes in the tree structure for each of the three rounds. One thing we should pay attention to is the change in the nodes in the hotspot areas (the hatched nodes in Figure 3). The node that was in a hotspot around the cluster head (hatched nodes in Figure 3a) was used as a general member node outside the hotspot area, as shown in Figure 3b, following a change in the cluster head. Similarly, the tree structure in the cluster, as seen in Figure 3b, is reconstructed after one round (i.e., after the mobile sink has visited), and it changes as shown in Figure 3c. As the new hotspot area around the head in this round is at a large distance from the hotspot area of the previous round, the nodes in the previous hotspot area have time to recover their energy.

### 3.4. Recharging Energy to the Cluster Head

**Figure 4:**
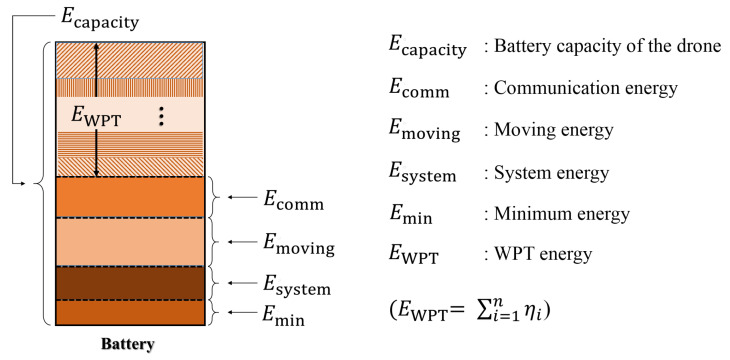
Energy model of the drone.

Before the mobile sink departs, the base station calculates the shortest travel path for the mobile sink to travel and the amount of energy that it can transmit to each cluster head. The shortest path is calculated with the TSP (traveling salesman problem) algorithm [33] by using the location information of the head, which was collected by the mobile sink in the previous round. Then, the mobile sink determines the amount of energy $E_{i}^{\mathrm{charge}}$ that it will give to the cluster head *i* on this shortest path during this visit. Figure 4 depicts the energy model of the drone. Assuming $E_{\mathrm{WPT}}$ is the available energy that a drone can distribute to nodes using WPT as described in Figure 4, $E_{i}^{\mathrm{charge}}$ can be calculated as follows:

(4) $$\begin{array}{cc} E_{i}^{\mathrm{charge}}\; & =\eta_{i}\times E_{\mathrm{WPT}}\\ \; & =\eta_{i}\times \left(E_{\mathrm{capacity}}-\left(E_{\mathrm{moving}}+E_{\mathrm{comm}}+E_{\mathrm{system}}+E_{\min}\right)\right),\\ \eta_{i} & =\bar{E_{\mathrm{header}}^{\mathrm{extra}}}/\sum_{k=1}^{n}\bar{E_{{\mathrm{header}}_{k}}^{\mathrm{extra}}}. \end{array}$$

Here, *i* is the index of the relevant cluster, *n* is the total number of clusters in the network, $E_{\mathrm{moving}}$ is the amount of energy used by the drone to move, $E_{\mathrm{capacity}}$ is the total battery capacity of the drone, $E_{\mathrm{comm}}$ is the amount of energy that the drone needs to collect data, $E_{\mathrm{system}}$ is the energy required for the complete drone system, and $E_{\min}$ is the minimum amount of energy required for the drone’s return. $\eta_{i}$ is the ratio of the average energy that the cluster head *i* has consumed until the current round to the average amount of energy that all cluster heads have consumed until the current round. This means that the mobile sink distributes the WPT energy ($E_{WPT}$) to the cluster head *i* according to the average amount of energy it consumed in cluster *i*. For example, if a cluster has a large number of member nodes, the energy consumed by its head may be greater than the energy consumed by the heads of other clusters. In such a case, the cluster head should be charged with a relatively large amount of energy.

Just before the completion of one round, the mobile sink visits the cluster head and performs two operations. (1) It receives the data collected throughout the cluster during this round from the head node, the head node information for the next round, and the value of $\bar{E_{\mathrm{header}}^{\mathrm{extra}}}$. (2) In addition, it charges each cluster head according to the amount obtained from Equation (4) to meet the ENO requirements.

### 3.5. Temporary Change of the Cluster Head

**Figure 5:**
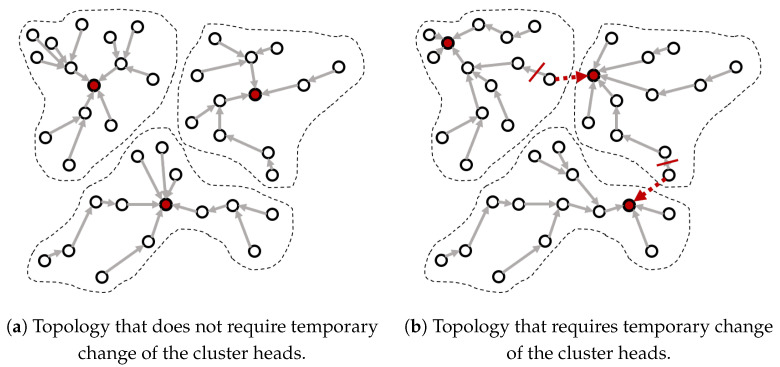
Temporary change of the cluster heads during the current round.

Figure 5 shows the way a node uses its energy efficiently by temporarily changing its cluster head when another cluster head is its neighboring node. The node transmits data to another cluster head if another cluster head is located within its one-hop distance, instead of transmitting data to its parent node. In the case of Figure 5a, since there is no node that is one-hop away from another cluster head, the data is transmitted through the original tree structure constructed in the setup phase. Conversely, in the case of Figure 5b, some nodes marked in red can reach another cluster head directly (with one-hop), and thus transfer their sensory data to the neighboring cluster heads instead of their parent nodes. This process is more likely when the cluster head is located near the boundary of the cluster as shown in Figure 5b. Through this approach, the relaying burden of the network can be reduced.

If the cluster configuration would be changed permanently (instead of temporarily for this round), it is impossible to maintain the cluster structure close to optimal as found by the harmony search algorithm, which results in a more severe energy imbalance between clusters and more blackout nodes.

### 3.6. Considerations for the Head Election Parameters

**Figure 6:**
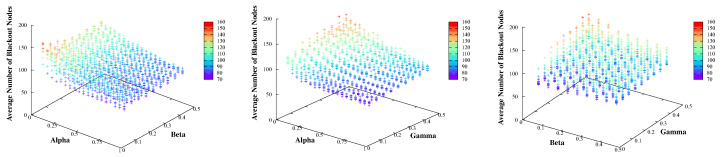
The average number of blackout nodes based on α, β, and γ.

Equation (3) is used to elect the best cluster head from among the candidate nodes that have sufficient energy to operate as head nodes. In this section, the effects of the parameters (α, β, and γ) on the head node selection are analyzed empirically. Figure 6 shows the average number of blackout nodes observed with different parameter values to determine the relationship between them. The number of blackout nodes is directly related to the performance of the proposed scheme.

As described in Table 3, the value of α is the weighting factor provided for controlling the importance of EV. As the proportion of α increases, a candidate head with less energy variance than its neighboring nodes has a higher possibility of being selected as the next cluster head. In Figure 6, the average number of blackout nodes generally decreases and then increases slightly for a short period near the end as the α increases. We confirmed that α was the most crucial parameter for reducing the number of blackout nodes. As only a node with sufficient energy (satisfying Equation (2)) can be a candidate head node, the small energy variation between the candidate node and its neighbors implies that the neighbor nodes also have sufficient energy. These energy-rich neighbors will become a part of an energy hotspot area if this candidate node is elected as the head. Therefore, these nodes can operate normally without experiencing blackouts even when they are located in a hotspot, leading to a better network-wide performance. However, if α is too high (i.e., only the energy variation is considered), a deterioration in performance is observed. Therefore, other parameters should also be considered.

The value of β is used to control the importance of the number of nodes located at one-hop distance from the candidate head. With an increase in β, the candidate node with more neighboring nodes has a larger possibility of being selected as the next cluster head. In the case of β, as shown in Figure 6, a high number of neighboring nodes around the candidate node is not always desirable. It may be better to have only the appropriate number of neighboring nodes according to the values of EV and HC in Equation (3).

The value of the γ factor controls the importance of the distance to the current head, and indicates that with an increase in the γ proportion, the candidate node that is furthest away from the current head has the highest possibility of being selected as the next head. As shown in Figure 6, choosing a candidate node with a smaller energy variation as the next cluster head is preferable, regardless of the distance from the current head node. In other words, it is not always desirable to have a large distance between the candidate and the current cluster head. Selecting a candidate farther from the current head is helpful to the network performance only when the energy imbalance becomes severe, since it then allows sufficient recovery time for the nodes in the previous hotspot. Consequently, the optimal value of γ varies depending on the cluster topology.

## 4. Performance Evaluation

To analyze the performance of the proposed method, we performed a simulation using Solar Castalia [34] modified for use as a mobile sink with WPT. The simulation parameters were set based on the experimental results of eZ430-RF2500 [35], a sensor node capable of harvesting energy, and a drone DJI Mavic Air model [36].

### 4.1. Simulation Environment

In the simulation, we compared the proposed scheme with other schemes, such as constructing clusters and finding the cluster heads (1) by considering the cohesion between the sensor nodes and the distance between the clusters(HSA-WSN) [13], (2) by taking only the energy into account (E-LEACH) [26], and (3) by considering multiple factors including its residual energy, number of hops to a base station, and number of neighboring nodes (HEEL) [27]. We deployed 400, 1000, and 1600 nodes in an area of 100 m × 100 m, and conducted experiments for 30 days of simulation time. All four schemes used the same solar-powered energy model, and the mobile sink charged the cluster heads in every round, which implies that the main differences among them are the method of clustering management, including the head election method, and the method of mobile sink management, including the determination of visiting path and the amount of charging energy transmitted to each head. In addition, the average amount of harvested energy measured by the real sensor node [35] was used, and the most appropriate α, β, and γ ratios were selected from among the results given in Figure 6 obtained after various experiments. Table 4 shows the detailed experimental environment.

### 4.2. Analysis of Experimental Results

**Figure 7:**
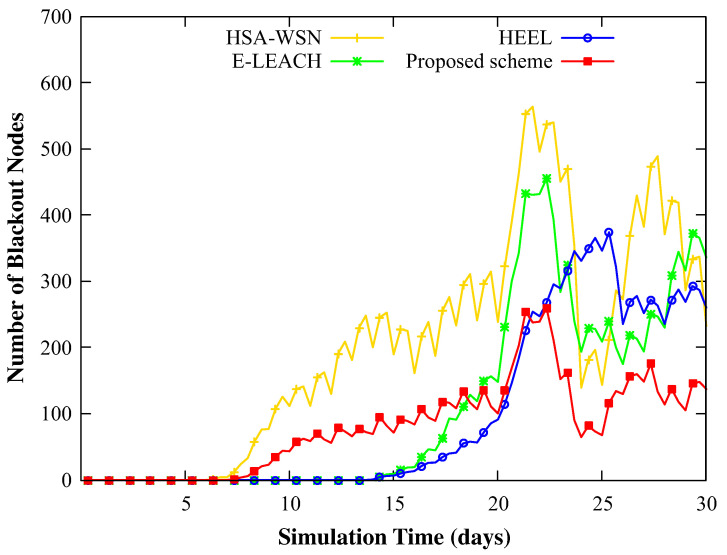
The change in the number of blackout nodes when simulating with 1600 nodes (21st–22th: rainy, 26th–27th: cloudy, and other: sunny).

Figure 7 shows the number of nodes that experienced blackouts over time among 1600 sensor nodes. HSA-WSN reported the first blackout node and the growth rate of the blackout nodes gradually increased. However, this scheme occasionally showed better performance than other schemes by selecting more appropriate nodes as the cluster heads. E-LEACH considered only its amount of residual energy for the election process (did not consider the energy status of neighboring nodes), and thus chose the node with the largest residual energy. Therefore, we observed that the number of blackout nodes increased sharply in this scheme as the energy imbalance became severe owing to the low residual energy of the neighboring nodes or low node density. HEEL showed better results than HSA-WSN and E-LEACH because it reflected the energy of itself and neighbors and the location information to manage the clusters. However, in many cases, the mobile sink could not visit all cluster heads since the number of clusters could not be controlled. As a result, there were many cluster heads that could not be charged from the mobile sink, and thus the number of blackout nodes occurs more than our scheme.

However, in the proposed scheme, despite showing results similar to other schemes in the early phase, the number of blackout nodes started to deviate over time after 11 days. This is because, in the beginning, the area with a small energy variance between the nodes was most likely the area with a high average energy, and thus a node near such a location is more likely to be selected as the next cluster head. However, over time, areas with small-energy-variance nodes start differing from areas with high-average-energy nodes due to energy imbalances. This results in the different number of blackout nodes. As for the E-LEACH, since the high energy of a head node does not translate to a balanced residual energy in all the neighboring nodes, much more blackout nodes were observed than in the proposed scheme. In the proposed scheme, temporary head change is applied according to the configured topology, which is one of the reasons that our scheme performs well.

**Figure 8:**
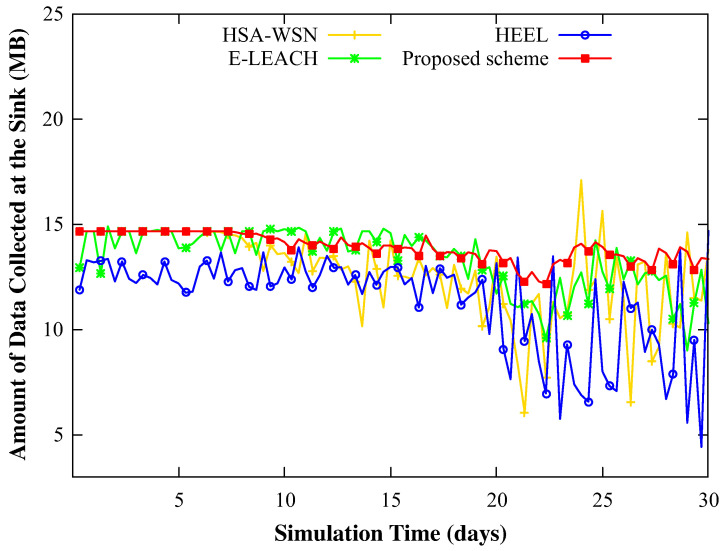
The change in the amount of data sensed during 30 days when simulating with 1600 nodes (21–22th: rainy, 26–27th: cloudy, and other: sunny).

Figure 8 shows the amount of data collected by the mobile sink over time from 1600 sensor nodes. In HEEL, the mobile sink could not visit all cluster heads since it construct so many clusters. As a result, mobile sink collects the least amount of data among four schemes. In HSA-WSN, the results confirmed that there was a large fluctuation in the amount of data collected by the mobile sink, depending on the distance of the head from the initial blackout node. The proposed scheme collected the largest amount of data. The results confirmed that the proposed scheme could collect the largest amount of data at the mobile sink. We think there were two reasons for this result. The first reason was that the blackouts occurred mainly at locations that were not fatal to the performance (i.e., locations other than the hotspot area around the head), when compared with other schemes. This is because the proposed scheme considered not only the residual energy but also the energy variance and density of the neighboring nodes. The second reason is that the mobile sink can visit all of the cluster head to collect data and charge the appropriate amount of energy.

This observation was more conspicuously applicable with the increasing severity of the weather. On a rainy day, due to the blackout of several nodes, the collected data of the working nodes was not relayed to the head. Therefore, it was observed that the amount of data gathered by the head increased sharply in clear weather for the other three schemes. However, the proposed method exhibited relatively small differences in the results in different weather conditions.

**Figure 9:**
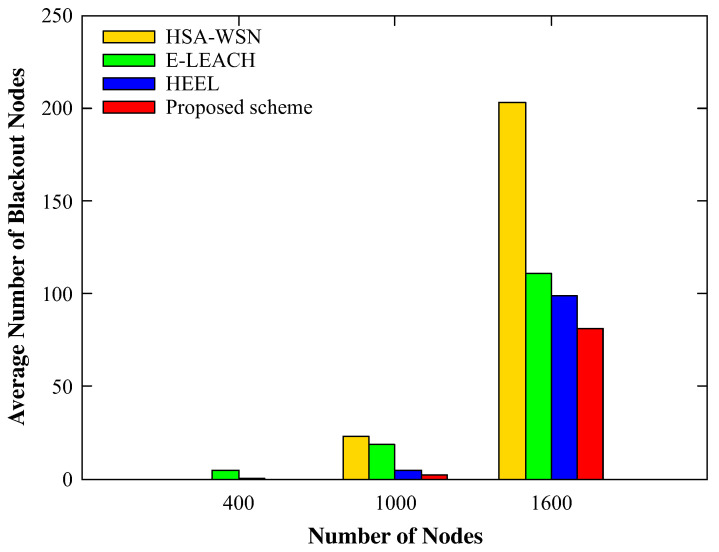
The number of blackout nodes according to the number of nodes.

**Figure 10:**
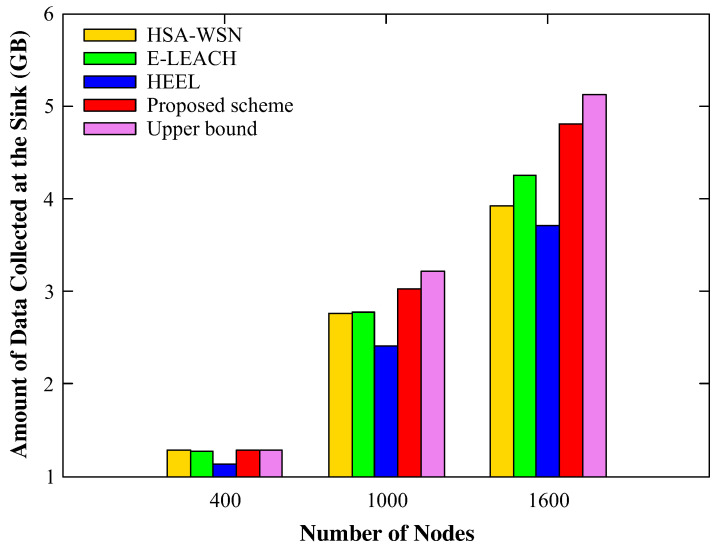
The amount of gathered data according to the number of nodes.

Figure 9 and Figure 10 show the average number of blackout nodes and the total amount of data gathered by the mobile sink based on the changing number of nodes. For 400 nodes, all four schemes showed similar results. The reason is that, as the number of sensor nodes in each cluster was not large, the amount of data did not exceed the processing and transporting capability of each node. Therefore, despite the relatively numerous data hotspots in the cluster, the mobile sink collected all the data accumulated in the head as the energy consumption was not high enough to make the sensor nodes black out. Thus, there were no blackout nodes when the density of the sensor nodes was low, regardless of the applied scheme. However, as the number of nodes increased, the amount of data that each node had to process and relay increased, leading to severe energy imbalances.

The HSA-WSN and E-LEACH without considering the energy imbalance showed the worst result in terms of blackout nodes, whereas HEEL and the proposed scheme that considered not only the energy but also various other factors reported the better result. However, since HEEL manages the cluster without considering the movement distance of the mobile sink, it yields the worst amount of data collected at the mobile sink. To sum up, we confirmed that the proposed scheme showed the best performance in all aspects, and especially, we could check that the amount of data collected at the mobile sink using the proposed scheme is closest to the theoretical upper bound calculated in the Appendix A.

By performing experiment while varying the number of nodes, we confirmed that our scheme worked without any problems, even with a very large number of nodes. In addition, as the number of nodes increased, the differences between the performance of the proposed scheme and those of other schemes became apparent.

## 5. Conclusions

In this study, for SP-WSNs that can operate permanently, we aimed to minimize the data loss due to blacked out nodes and also to ensure the maximum possible nodes achieved ENO by using an efficient clustering and head election scheme as well as a wireless energy charging scheme for the mobile sink. The proposed scheme achieved close-to-optimal clustering using HSA in the base station. Next, the WPT-capable mobile sink simultaneously collected the data and charged the cluster heads with its own energy, thereby preventing any lack of energy in the head nodes. In addition, the proposed scheme provided sufficient time for the previous energy hotspot nodes to fully recover their energy through careful determination of the next cluster head among the nodes with sufficient energy, thereby solving the hotspot problem. Consequently, the proposed scheme gathered more data than the other schemes, and also achieved higher efficiency as the number of nodes increased.

## Figures and Tables

**Table 1:** The power densities of different energy sources [14].

Energy Source	Types	Energy Harvesting Method	Power Density
Radiant	Solar	Solar cells	15 mW/cm${}^{2}$
	Wind Flow	Electromechanical conversion	16.2 μW/cm${}^{3}$
Mechanical	Acoustic Noise	Piezoelectric	960 nW/cm${}^{3}$
	Motion	Piezoelectric	330 μW/cm${}^{3}$
Thermal	Body heat	Thermoelectric	40 μW/cm${}^{2}$

**Table 2:** Comparison of mobile sinks.

	Energy	Mobility	Automation	Cost
**Vehicle**	Infinite	Limited	Unable	High
**UAV**	Finite	Unlimited	Able	Low

**Table 3:** Weighting factors for the cluster head election.

Weight	Content
α	Energy variance between candidate head and neighbor nodes (EV)
β	Number of neighbor nodes within one-hop distance (NN)
γ	Hop count between candidate head and current head (HC)

**Table 4:** Simulation parameters.

Parameter	Value
Simulation time	30 days
Field size	100 m × 100 m
Number of nodes	400, 1000, 1600
Deployment	Random
Weather	Randomly selected (sunny, cloudy, or rainy)
Sensor node battery capacity	100 mAh
Min./Avg./Max. harvesting energy per day	19.69J/46.59 J/49.22 J
Sensor nodes sensing data	80 bytes/min
Sensor nodes transmission range	10 m
*TX* energy	0.9313 nJ/byte
*RX* energy	0.1891 μJ/byte
Mobile sink battery capacity	2375 mAh
WPT efficiency	50%
α:β:γ	0.8:0.1:0.1

**Figure A1:**
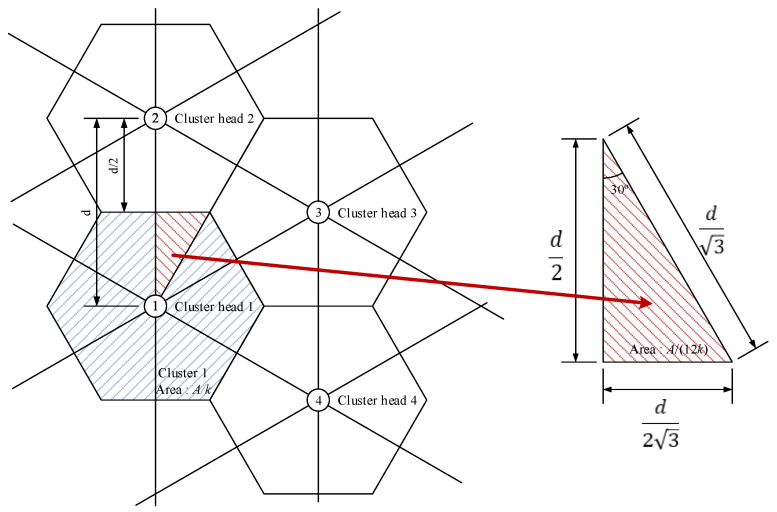
Cluster structure.

## Appendix A. Estimation of Upper Bound of the Amount of Data Collected

In this paper, we compared the performance with the upper bound of the amount of data collected to evaluate the proposed scheme. In this appendix, we introduce how to find the upper bound we used.

In the proposed scheme, the solar-powered sensor nodes form clusters, the sensor nodes deliver the sensed data to the cluster heads, and, each round, the mobile sink traverses the cluster heads to collect data. At this time, the mobile sink transfers energy to the cluster heads. To find the upper bound of the amount of data collected in this environment, we made assumptions as follows.

(i) $n_{}$ nodes are evenly distributed in a field of size *A*.
(ii) There are $k_{}$ clusters and the optimal clusters are found (the area is divided equally).
(iii) The energy delivered by the sink node is distributed equally among all nodes to balance the energy.

To calculate the upper bound in this environment, it is necessary to find the energy consumed by all nodes, the amount of solar energy harvested, and the amount of energy delivered by the mobile sink node.

### Appendix A.1. Energy Consumed by All Sensor Nodes

The energy ${E_{}}_{c}^{\mathrm{round}}$ consumed by $n_{}$ for 1 round is as follows:

(A1) $${E_{}}_{c}^{\mathrm{round}}={E_{}}_{c}^{\mathrm{idle}}+{E_{}}_{\mathrm{Tx}}^{\mathrm{round}},$$

where ${E_{}}_{c}^{\mathrm{idle}}$ is the energy that the nodes consumes fixedly, excluding data transmission, and ${E_{}}_{\mathrm{Tx}}^{\mathrm{round}}$ is the transmission energy. In order to calculate ${E_{}}_{\mathrm{Tx}}^{\mathrm{round}}$, the number of data transmissions of the sensor node should be obtained first.

#### Appendix A.1.1. The Number of Nodes According to the Distance from the Cluster Heads

All sensor nodes periodically collect data and deliver them to their cluster heads in a multi-hop manner. Therefore, the number of transmissions for data of each node to reach the cluster head depends on how many hops there are from the cluster heads.

First, we calculate the number of nodes according to the distance in a cluster. We calculate the number of nodes in the transmission range $t_{}$ of a cluster head using the area of the $t_{}$ range and the node density γ. In other words, because there are, on average, aγ nodes in the area of *a*, the area in the $t_{}$ range is calculated to obtain the number of nodes. The number of nodes at the one-hop distance $n_{1}$ is calculated as follows:

(A2) $$n_{1}=\min\left(n_{c},\gamma \pi {t_{}}^{2}\right)-1,$$

where $n_{c}$ is the total number of nodes in the cluster. The number of nodes less than $n_{c}$ within the range of $t_{}$, excluding the cluster head, is obtained. Within the range of $t_{}$, the number of nodes less than or equal to $n_{c}$ is calculated excluding the cluster head. The number of nodes $n_{i}$ corresponding to the *i* hop can be calculated as follows:

(A3) $$\begin{array}{cc} n_{2} & =\min\left(n_{c},\gamma \pi {\left(2t_{}\right)}^{2}\right)-n_{1}-1\\ n_{3} & =\min\left(n_{c},\gamma \pi {\left(3t_{}\right)}^{2}\right)-\left(n_{1}+n_{2}\right)-1\\ n_{4} & =\min\left(n_{c},\gamma \pi {\left(4t_{}\right)}^{2}\right)-\left(n_{1}+n_{2}+n_{3}\right)-1\\ \; & \ldots \\ n_{i} & =\min\left(n_{c},i^{2}\gamma \pi {t_{}}^{2}\right)-\sum_{j=1}^{i-1}n_{j}-1. \end{array}$$

#### Appendix A.1.2. Transmission Energy of All Sensor Nodes

When all nodes in a cluster transmit data, the transmissions are equal in number to the hops that are needed for the data to reach the cluster head. Therefore, the number of transmissions performed in a cluster is $\sum_{i=1}^{i_{\max}}n_{i}i$. Each transfer consumes ${E_{}}_{\mathrm{Tx}}$ energy, and if there are $k_{}$ clusters, the consumed energy ${E_{}}_{\mathrm{Tx}}^{\mathrm{once}}$ when all nodes transmit data once is as follows:

(A4) $${E_{}}_{\mathrm{Tx}}^{\mathrm{once}}={E_{}}_{\mathrm{Tx}}k_{}\sum_{i=1}^{i_{\max}}n_{i}i.$$

The transmission energy ${E_{}}_{\mathrm{Tx}}^{\mathrm{round}}$ consumed in the entire network for $m_{\mathrm{Tx}}$ data transmission per round is as follows:

(A5) $${E_{}}_{\mathrm{Tx}}^{\mathrm{round}}=m_{\mathrm{Tx}}{E_{}}_{\mathrm{Tx}}^{\mathrm{once}}+m_{\mathrm{Tx}}n_{}{E_{}}_{\mathrm{Tx}},$$

where $m_{\mathrm{Tx}}n_{}{E_{}}_{\mathrm{Tx}}$ is the energy consumed when the head nodes transmit data to the mobile sink node. By substituting ${E_{}}_{\mathrm{Tx}}^{\mathrm{round}}$ into Equation (A1), the total energy consumption of the nodes can be calculated in one round.

### Appendix A.2. The Energy Charged to Nodes

#### Appendix A.2.1. The Amount of Solar Energy Harvested

The amount of harvested solar energy depends on the time and weather. The amount of solar energy ${E_{}}_{h}^{\mathrm{round}}$ that all nodes collect during one round can be calculated hourly through harvested energy estimations, such as pro-energy [37].

#### Appendix A.2.2. Amount of Chargeable Energy of the Mobile Sink

The energy model of the mobile sink used in this article is as follows:

(A6) $${E_{}}_{\mathrm{charge}}=E_{\mathrm{capacity}}-\left(E_{\mathrm{moving}}+E_{\mathrm{comm}}+E_{\mathrm{system}}+E_{\min}\right),$$

where the battery capacity is $E_{\mathrm{capacity}}$, communication energy is $E_{\mathrm{comm}}$, idle energy is $E_{\mathrm{system}}$, and minimum energy is $E_{\min}$, and they are fixed every round. Therefore, the amount of chargeable energy ${E_{}}_{\mathrm{charge}}$ is determined according to the moving energy ${E_{}}_{\mathrm{move}}$ of the mobile sink.

The mobile sink moves from one cluster head to another. Thus, first, we need to find the distance $d_{}$ between cluster heads. We use a hexagonal shape arrangement known to be efficient [38] in terms of load balancing and energy distribution, such as Figure A1. When the area of one cluster is $A/k_{}$, and the area of the right triangle in Figure A1 is ${d_{}}^{2}/\left(8\sqrt{3}\right)$, the distance between each cluster head is $d_{}$, which can be calculated as follows:

(A7) $$d_{h}=\sqrt{\frac{2\sqrt{3}}{3}\frac{A}{k}}.$$

When the mobile sink moves to each cluster head, it must move $d_{}$, the distance between cluster heads and move $k_{}-1$ times. When it moves in and out of the network at the beginning and end of the traverse, respectively, it moves at least $k_{}/2$ each. Therefore, the total moving distance ${d_{}}_{\mathrm{move}}$ is as follows:

(A8) $$\begin{array}{cc} \; & {d_{}}_{\mathrm{move}}=\left(k_{}-1\right)d_{}+k_{}/2d_{}+k_{}/2\left(k_{}-1\right)d_{} \end{array}$$

(A9) $$\begin{array}{cc} \; & =k_{}d_{}. \end{array}$$

By substituting ${d_{}}_{\mathrm{move}}$ into Equation (A6), we can obtain the amount of chargeable energy ${E_{}}_{\mathrm{charge}}$ during one round.

### Appendix A.3. The Upper Bound of the Data Collected

In order to calculate the upper bound of the data collected, since we assumed that all chargeable energy was distributed equally, as is necessary to the node, and that the available energy was perfectly balanced among all nodes, in the case that the available energy is insufficient, all nodes cannot transmit data at the same time. We calculate the upper bound of the data collection during the day using this property.

The total energy gained by the entire network during one round is as follows:

(A10) $${E_{}}_{\mathrm{charge}}+{E_{}}_{h}^{\mathrm{round}},$$

and the energy for one day is as follows:

(A11) $$m_{\mathrm{round}}{E_{}}_{\mathrm{charge}}+{E_{}}_{h}^{\mathrm{day}}.$$

Therefore, when the total amount of energy that the nodes currently have is ${E_{}}_{\mathrm{remain}}^{\mathrm{total}}$, the amount of available energy ${E_{}}_{\mathrm{avail}}$ for a day is as follows:

(A12) $${E_{}}_{\mathrm{avail}}=m_{\mathrm{round}}{E_{}}_{\mathrm{charge}}+{E_{}}_{h}^{\mathrm{day}}+{E_{}}_{\mathrm{remain}}^{\mathrm{total}}.$$

Therefore, the upper bound $s_{upper}$ of data gathered when ${E_{}}_{c}^{\mathrm{round}}$ is consumed every round within the available energy ${E_{}}_{\mathrm{avail}}$ can be calculated as follows:

(A13) $$s_{upper}=n_{}m_{\mathrm{Tx}}\min\left(\left\lfloor \frac{{E_{}}_{\mathrm{avail}}}{{E_{}}_{c}^{\mathrm{round}}}\right\rfloor ,m_{\mathrm{round}}\right).$$
